# The Limits of Our Explanation: A Case Study in *Myxococcus xanthus* Cooperation

**DOI:** 10.1007/s13752-024-00479-z

**Published:** 2024-11-12

**Authors:** Saira Khan

**Affiliations:** Department of Philosophy, University of Bristol, Bristol, UK

**Keywords:** Cooperation, Microbiology, Myxobacteria, *Myxococcus xanthus*, Predation, Public Goods game

## Abstract

In this article, I demonstrate two ways in which our major theories of the evolution of cooperation may fail to capture particular social phenomena. The first shortcoming of our current major theories stems from the possibility of mischaracterizing the cooperative problem in game theory. The second shortcoming of our current major theories is the insensitivity of these explanatory models to ecological and genomic context. As a case study to illustrate these points, I will use the cooperative interaction of a species of myxobacteria called *Myxococcus xanthus. M. xanthus* cooperate in many areas of their life cycle—in quorum sensing, social motility, fruiting body formation, and predation. I focus in particular on predation as we have not yet discovered an adequate explanation of how they sustain cooperative predation in the face of developmental cheats. In explaining why we have not, I draw generalizable conclusions that shed light on our use of simplified models to explain real-world behaviors in a variety of organisms.

## Introduction

Biologists have long been concerned with how cooperative behavior could have arisen when cooperation is costly and natural selection favors those traits which contribute to reproductive success. In order to understand the problem, we must first be clear on what we mean by cooperation. Hamilton (1964) famously classified social behavior into four types based on the fitness consequences for the actor and recipient in an interaction, as shown in Table 1. Whether a behavior is beneficial or costly depends on the lifetime fitness consequences of the behavior and the absolute fitness effect.^1^

**Table 1:** Hamiltonian classification of social behaviors.

Effect on actor	Effect on recipient	
	+	-
+	Mutual benefit	Selfishness
-	Altruism	Spite

In what follows, I will primarily refer to *interactions* or *outcomes* in which both agents benefit as situations of “mutual benefit,” rather than referring to the *behaviors* or *actions* of a particular agent as mutually beneficial as in Hamilton’s schema. This is because, if an action is straightforwardly beneficial to an agent, it does not require explanation—the behavior or trait which contributes to such behavior would be selected for. The puzzle in the case of altruistic behavior is that it is costly to one’s fitness while natural selection favors traits that contribute to reproductive success. The puzzle in instances where we are concerned with a mutually beneficial outcome is that it may be profitable for an individual to free ride or to cheat to reduce her own costs, and we would expect the actor to do so since this secures better relative fitness consequences.

While many existing accounts of the evolution of cooperation focus on explaining altruism—behaviors that are costly to the actor—cooperation also includes those behaviors that are mutually beneficial. Indeed, in the ordinary sense of the word, we often take cooperation to refer to collaborative action to achieve a joint goal. The terms “altruism” and “cooperation” have been used differently in different parts of the social evolution literature (West et al. 2006, 2007). I follow the work of Sachs et al. (2004) in using cooperation to refer to those social behaviors that provide a benefit to the recipient but may be *either* beneficial or costly to the agent. This covers cases of both altruism and mutual benefit since, as we will see, cooperation in myxobacteria may be of both sorts.

Despite the puzzles presented above, we do see stable cooperation for altruistic and mutually beneficial behavior in both the animal kingdom and among single-celled organisms. Many theories have been offered to explain this, including but not limited to, kin selection (Hamilton 1963), group selection (Sober and Wilson 1998), reciprocal altruism (Trivers 1971), punishment (Boyd and Richerson 1992), pre-play signaling (Robson 1990), indirect reciprocity (Alexander 1987) and commitment (Schelling 1960).^2^ Theories which appeal to *direct* benefit to the cooperator—such as reciprocal altruism, pre-play signaling, and commitment—can explain cooperation where cooperation is mutually beneficial. Theories which appeal to *indirect* benefits—kin selection and, sometimes, group selection—can explain cooperation where cooperation is genuinely costly or altruistic for the agent.^3^

The effectiveness of such theories in adequately accounting for social phenomena has been questioned. Tinbergen (1963) showed that there are different ways behavior can be explained, including focus on mechanisms (causal explanations) and evolutionary consequences (functional explanations). The aforementioned theories are functional explanations: they explain cooperative behavior by appeal to the fitness consequences of such behaviors without necessarily looking to the proximate mechanisms—physiological, chemical, or psychological—that cause them. Some have argued that purely functional accounts of behavior are inadequate, and so call for an integration of function and mechanism (Real 1993; McNamara and Houston 2009; McNamara and Leimar 2019). This objection is put forth most clearly in the field of behavioral ecology, which often makes assumptions that organisms can respond flexibly by adopting the optimal behavior for each circumstance. However, as ethologists have made clear, behavior can be determined by mechanisms that do not exhibit optimal behavior in every circumstance. Thus, functional explanations devoid of biological realism and mechanistic detail are lacking in their ability to account for real-world social behaviors.

There is also a more general difficulty in applying abstract, how-possibly theories to real-world phenomena. This is true of scientific inquiry even outside the domain of biology; the use of model-based science has been much debated (Orzack and Sober 1993; Odenbaugh 2003, 2006; Godfrey-Smith 2006, 2009; Potochnik 2007; Matthewson and Weisberg 2009; Knuuttila 2011; Matthewson 2011; Weisberg 2013). Indeed, Levins (1966) famously noted a trade-off between generality, realism, and precision. Type 1 models are those which include as many parameters as possible and so generate precise, testable outputs. Here, generality is sacrificed for realism and precision. Type 2 models are those which provide general equations that generate precise outputs but involve unrealistic idealizations and assumptions. They sacrifice realism for precision and generality. Type 2 models may be those which only represent part of a system. They are thereby general and avoid unrealistic examples, but sacrifice precision or testability. The theories of the evolution of cooperation I mentioned earlier might be thought of as Type 3 models—those which prefer generality and realism over precision—though, as we will see, the extent to which realism is represented is also debatable.

Despite these critiques of the theories, they have not lost prevalence as the framework within which we generally seek to explain cooperation. In this article, I seek to draw further attention to the limitations of applying these coarse-grained theories to real-world phenomena. I highlight two limitations. The first limitation is the potential for a mischaracterization of the game. That is, our major theories of cooperation mentioned above do not offer insights into the representation of any particular cooperative interaction in terms of game theory. They offer explanations for cooperation once the game has already been given and the explanation will change depending on the game under consideration. For example, whether we characterize a particular social phenomenon as a Prisoner’s Dilemma or a Stag Hunt will change whether we believe the agent has an incentive to defect or whether they simply require credible signaling to ensure cooperation. In the Prisoner’s Dilemma, a credible signal to cooperate does not induce cooperation by the other player, since it pays to defect if one’s partner cooperates. In the Stag Hunt, credible signaling will be sufficient to ensure mutual cooperation. The particular issue I will be focusing on is the way in which we characterize public goods games.

The second limitation is the dependence of cooperation on ecological and genomic context. Many of our major cooperative models are not fine-grained to capture the effect of the natural environment, the interaction of genotype and environment, or the potential for transient phases of cooperation or defection. Each of these considerations will affect whether an organism exhibits cooperative or defective behavior, so paying heed to these contextual details may change our explanation of the social phenomenon at hand. For example, the same organisms in a nutrient-rich environment may be less cooperative than they are in a nutrient-sparse environment. As a toy example, imagine a pack of wolves in a zoo enclosure who are fed regularly versus those whose only access to food is available via a group hunt. The latter may exhibit more cooperativeness due to the nature of their environment but would subsequently change their behavior if they were to find themselves in a nutrient-rich environment. Indeed, there may even be conflict. Characterizations of behaviors as simply cooperative or defective obscures the important fact of what prompts this cooperative behavior, the conditions under which it may change, and the ways in which phenotypic expression of cooperative behavior are linked to both genotype and environmental context.

I use the myxobacteria species *Myxoccocus xanthus* as a case study to elucidate the limitations of these theories as it offers a paradigm case for studying the evolution of cooperation more generally. Myxobacteria are bacteria which typically live in soil and feed on insoluble inorganic substances (Furness et al. 2020). They are one of the bacterial groups that have transitioned from single cell to multicellular life, engaging in varied cooperative behaviors comparable in sophistication to that seen in macroscopic social organisms (Muñoz-Dorado et al. 2016). The species *Myxococcus xanthus* has received notable attention. It demonstrates multifaceted social behaviors and flexible use of resources to ensure survival by adopting a multicellular lifestyle when required. It cooperates in several areas of its developmental life cycle. First, *M. xanthus* cells cooperate in social motility or swarming. Second, they cooperate in the formation of spore-producing fruiting bodies. Third, they cooperate in predation. All forms of cooperation (swarming, fruiting body formation, and predation) arguably involve a fourth form of cooperation, quorum sensing, since these activities depend on high cell densities. “Quorum sensing” is a term that refers to the communication between bacterial cells, via the production of diffusible molecules, to achieve a group-level response. Furthermore, multicellularity in myxobacteria is not obligatory, but rather transitory, allowing us to further investigate the conditions for the evolution of these distinctive forms of cooperation (António and Schulze-Makuch 2012). By studying *M. xanthus*, researchers can gain insight into the emergence of complex multicellular structures and the mechanisms that underlie this, with consequences for how we study the evolution of cooperation more generally. I focus in particular on *M. xanthus* cooperative predation as this is a social phenomenon that we currently do not have a good theory for, though the criticisms raised here will also apply to other cooperative behaviors.

Indeed, the focus on cooperative predation does not imply that ecological context and characterization of the game do not matter in explanations of *other M. xanthus* cooperative activities. This is a general point about the limitations of such coarse-grained explanatory models in capturing any social phenomena. I use group hunting as the illustrative example since this is a paradigm case of difficult-to-explain cooperative behavior and the mechanisms sustaining cooperation are less well-understood. I seek to show why this might be, by illustrating the difficulties of applying coarse-grained explanatory models to real-world phenomena and by drawing out where these difficulties are stemming from. Neither will I be claiming that our major theories *cannot* be operating to secure cooperation. Rather, their application is explanatorily inconclusive without further mechanistic detail. Fitting such models neatly onto *M. xanthus* cooperative predation is flawed as a result of a potential mischaracterization of the game and a lack of consideration of ecological and genomic context which complicate the picture. This will be true of many social phenomena. Bringing attention to this fact will help us to draw better conclusions about the explanatory scope of such theories of the evolution of cooperation as well as to understand where, and in what ways, they fall short.

In the second section, I show how kin selection, signaling, and punishment have been offered as explanations of cooperation in *M. xanthus* quorum sensing, social motility, and fruiting body formation. In the third section, I argue that these theories cannot seem to explain *M. xanthus* cooperative predation at first pass. Furthermore, other prominent theories appear similarly inadequate to explain cooperative predation. I offer two explanations for why this might be the case. First, cooperative predation might have been mischaracterized as a linear public goods game or a game in which selection is operating on strategies, not on genes which influence multiple strategies. Second, these theories of the evolution of cooperation are silent on the matter of ecological and genomic context which can change the expression of cooperation or defection, the strategy space, as well as the game being played. In section four, I summarize and discuss how these considerations ought to inform our use of simplified models to explain the evolution of cooperation in *M. xanthus*, as well as in other organisms more generally. In order to understand why *M. xanthus* social predation is difficult to explain, it will be instructive to first look at explanations proposed for their other cooperative behaviors.

## Explaining Cooperation in *Myxococcus xanthus*

### Kin Selection

Some have suggested that kin selection explains the cooperative stability of *M. xanthus* quorum sensing and fruiting body formation. Hamilton (1963) proposed an explanation of the evolution of altruism called the theory of inclusive fitness, relabeled kin selection by Maynard Smith (1964). Under kin selection, an organism favors the reproductive success of its relatives at a cost to itself, thereby acting altruistically. Hamilton theorized that altruism will occur where the affected individual is a relative of the altruist so has a greater chance of possessing the “cooperative gene.” Hamilton showed that if the gain to a relative of degree *r* is *k*-times the loss to the altruist, the criterion for positive selection of the gene is when *k* is greater than $\:\frac{1}{r}$. To elucidate, altruism among siblings will be selected only if the behavior produces twice the gain than loss. There is also a broader understanding of kin selection which emphasizes the relevance of individuals who share the same genes but do not possess common ancestry. In this case, indirect fitness benefits are realized through behaviors that positively impact nonrelatives with the cooperative gene. In some cases, kin *recognition* is required so cooperation can be preferentially directed to cooperators. In these cases, a single gene or a number of linked genes must cause both the cooperative behavior and be recognized by others through a phenotypic marker, known as the “greenbeard effect” (Hamilton 1964; Dawkins 1976).

Quorum sensing is an activity that precedes density-dependent activities, in particular *M. xanthus* social motility (Miller and Bassler 2001). Yet quorum sensing is itself a cooperative behavior in need of explanation. The reason we need an explanation is due to the problem of cheats. There are two ways in which bacteria could cheat in a quorum-sensing scenario. One possibility is that a cheater does not produce the quorum-sensing signal. It thereby benefits from the efforts of other cells in monitoring local cell density while avoiding the costs associated with signal production. An alternative possibility is that the actor overproduces the costly signal but does not respond to it. This can result in neighboring cells producing more of the public good while the actor does not (Diggle et al. 2007a). In light of the problem of cheats or free riders, the model proposed to explain the stability of quorum sensing in the *M. xanthus* literature is kin selection.

Brown and Johnstone (2001) apply Hamilton’s theory of kin selection to explain the stability of cooperative quorum sensing in bacteria. They find, via computer simulations, that the evolutionarily stable level of signaling and public goods production both increase with greater population density and with increased relatedness. Furthermore, with intermediate relatedness, there can still be selection to produce public goods but it is in the interests of each cell to produce less than others. As such, modelling results show kin selection may offer an explanation for cooperative quorum sensing. There is also empirical support for the kin selection hypothesis for quorum sensing from studies on other bacteria, in particular *Pseudomonas aeruginosa* (Diggle et al. 2007b). It was found that when quorum-sensing proficient cells were isolated from deficient cells and thereby relatedness was high, the relative fitness of the strain increases. Popat et al. (2015) also find that signaling decreases monotonically with relatedness in other quorum-sensing bacteria.

Brown and Johnstone’s model does not propose a mechanism behind kin selection but it is likely assumed to be operating by way of limited dispersal of related or cooperative individuals, such that one’s group of interactants consists of like kind. However, where limited dispersal is not guaranteed, we require some means of kin recognition—using information to discern which actors are also cooperators and to preferentially direct cooperation toward these actors. Indeed, cell diversity exists in myxobacterial populations which might necessitate kin recognition. Genetic diversity can be intensified by environmental stresses and mutations (Furness et al. 2020). Not only this, but genetically identical cells may differ phenotypically as a result of cellular wounds, aging, and starvation (Cao et al. 2015). Although such phenotypic variation would not affect kin selection per se—since the important aspect of kin selection is that cooperation is directed to *genetically* similar individuals—this may affect kin recognition. Phenotypic variation would make it difficult to detect like kind. As a result, some mechanism of ensuring that cooperative cells interact with other cooperative cells is required to ensure the stability of cooperative interaction.

Kin recognition, rather than kin selection, has been proposed to explain fruiting body formation among *M. xanthus.* Fruiting body formation is an instance of cooperation as it involves the aggregation of cells and division of tasks within the colony. One in every ten cells is likely to transform from its original rod-shaped form into a spherical spore that can withstand changes in temperature and desiccation. Three out of every ten cells will differentiate into peripheral rods that search for prey. Six out of every ten cells will be programmed to die and provide nutrients that assist in the morphogenesis of rod-shaped cells into spores. The division of labor seen in the different cells here is analogous to the various roles played by eusocial insects in colonies (Muñoz-Dorado et al. 2016). As mentioned earlier, cell diversity exists in myxobacterial populations and, as such, interacting with like kind is not assured. One mechanism suggested to overcome kin dispersal and facilitate kin discrimination in fruiting body formation has been the outer membrane exchange (OME) (Cao et al. 2015). The OME is a bidirectional transfer of outer membrane (OM) components such as lipids, lipoproteins, and lipopolysaccharides between two cells. OME dilutes damage to a few bacterial cells among the rest of the population and helps to restore mobility and sporulation. When a healthy population of *M. xanthus* cells was incapable of OME, increased cell density had no effect on sporulation outcomes, suggesting that cell density is only important when damaged cells can be repaired by OME (Cao et al. 2015). Studies have shown that such OME can be responsible for transfer of motility proteins to nonmotile mutant strains, making them temporarily motile (Hodgkin and Kaiser 1977; Velicer and Yu 2003).

Importantly, OME is achieved via the interaction of two surface proteins called TraA and TraB. First, they are responsible for bringing the two cells in close enough proximity for OME to occur. OME transfer requires motility in order to align cells and direct cell-to-cell contact. Second, they are responsible for ensuring OME only occurs between particular cells. This is because TraA proteins only recognize identical TraA proteins on other cells (Cao et al. 2015). As a result, *M. xanthus* cells engaging in OME are protected from accidentally aiding distantly related or competing *M. xanthus* populations. The TraA has a domain architecture and sequence similar to those of the FLO1 surface receptor which is required for flocculation in *Saccharomyces cerevisiae.* This is illuminating as the FLO1 sequence has previously been described as a “greenbeard” gene that ensures cooperation is directed towards other cooperators (Smukalla et al. 2008). As such, Cao et al. (2015) propose that TraA serves an analogous role in *M. xanthus* and note that TraA meets other conditions required of a greenbeard gene, for example, that it is polymorphic to allow specificity during recognition. Not only this, but in cases where cells express compatible TraA receptors, but are not siblings, outward swarm expansion is blocked by OME-mediated cell poisoning via lipoprotein toxins (Dey and Wall 2014; Dey et al. 2016).

So kin selection and kin recognition have been proposed to explain both myxobacteria quorum sensing, which precedes density-dependent activities, and fruiting body formation via TraA-dependent cell recognition in OME. Both of these explanations rely on cells being able to interact with cells with the relevantly similar genotype. In the case of quorum sensing, the proposed mechanism is likely limited dispersal. In the case of fruiting body formation, preferential interaction is achieved via protein recognition upon cell-to-cell contact.

### Signaling

Another mechanism by which cooperation can be stable despite the potential for cheats is through signaling. If agents can signal to one another that they are both cooperators before beginning an interaction, they can ensure that their interaction is preferentially directed toward other cooperators and thus avoid exploitation by cheats. It has been argued that signals are used in *M. xanthus* social motility.

*Myxococcus xanthus* have two types of motility, known as A-motility and S-motility. A-motility is observed for single cells and S-motility is when myxobacterial cells preferentially travel along slime trails left by other cells. Myxobacterial cells bind to exopolysaccharides either on neighboring cells or on the gliding surface. Therefore, S-motility requires that cells be in close proximity to achieve group swarming. The majority of cells in *M. xanthus* colonies are observed in tendril-shaped groups using this social motility, though isolated cells can be observed moving at the edges of the colonies. Social motility among *M. xanthus* cells is analogous to the social organization seen in ants, whose movement is mediated by antennae-borne chemosensory systems (Muñoz-Dorado et al. 2016). Ducret et al. (2013) discuss how slime-embedded OM materials or outer membrane vesicles (OMVs) could contain signals that allow for recognition of other cells and facilitate trail-following in *M. xanthus.* Furthermore, studies of rippling behavior, which is also a form of coordinated movement, indicate that these movements can be produced by side-to-side signaling between cells, causing cells to locally align (Igoshin et al. 2001, 2004; Sliusarenko et al. 2006; Zhang et al. 2012). It has been suggested that this rippling behavior increases the rate of colony expansion at the same time as reducing the mean square displacement of individual cells, allowing the *M. xanthus* population to be within the prey area longer (Berleman et al. 2008; Zhang et al. 2012).^4^

Signaling is also arguably seen in fruiting body formation. During the initial phases of fruiting body formation, *M. xanthus* cells accumulate elevated levels of (p)ppGpp in response to starvation (Harris et al. 1998; Pathak et al. 2012). This leads to both the activation of certain early developmental genes as well as the secretion of what’s known as the “A-signal.” This signal is composed of a set of peptides and amino acids that are released into the extracellular space by proteases (Kuspa et al. 1986, 1992; Plamann et al. 1992). The A-signal is a quorum-like signal believed to be a means of monitoring local cell density. As this signal reaches a threshold, ensuring quorum is reached, gene expression related to development of fruiting bodies is initiated. Whilst it seems like using nutrients to indicate nutrient scarcity may be maladaptive, the proposed benefit lies in the ability of the cells to recognize real situations of nutrient scarcity rather than responding to potentially dishonest signals from cheating genotypes (Whitworth et al. 2015). The explanation being proposed here is that of costly signaling.

The costly signaling hypothesis is not a theory of the evolution of cooperation but rather shows how signals can be honest in the face of potential deception. It was first proposed to account for difficult-to-explain behaviors such as bird mating displays. The suggestion is that these difficult-to-explain behaviors are a result of actors signaling their phenotypic quality to reproductive partners, benefitting the sender of the signal (Hawkes 1991). In the A-signal case, the proposed explanation is that intrinsic costs are involved in the production of the signal (the use of nutrients) which deter deceptive agents from issuing the signal. As a result, only honest agents signal and the signal is a reliable means of communication among cooperators.

So signaling is a proposed explanation for the cooperative stability of both motility and fruiting body formation. The explanation in the case of motility involves signaling via OM materials which allows recognition of other cells and facilitates trail-following, as well as side-to-side signaling between cells during rippling behavior. These signals ensure that cooperators interact with like types. The explanation in the case of fruiting body formation is the A-signal, which is initiated in response to elevated (p)ppGpp in response to starvation. The honesty of this signal is potentially ensured by its intrinsic cost in terms of nutrient usage, meaning deceptive agents are deterred from signaling.

### Punishment

A final mechanism that explains the stability of cooperation in the face of potential cheating is punishment.^5^ In laboratory experiments concerning fruiting body formation, it has been shown that continuous exploitation by cheaters (mutants in the gene *csgA*, which is important for cell–cell signaling in development) leads to the development of a cooperator strain that can police or punish the cheating genotype (Manhes and Velicer 2011). The mechanism of policing is not known but is believed to involve the production of an inhibitory agent. Indeed, Smith and Dworkin (1994) note the presence of bacteriocin and antibiotic mediated killing in *M. xanthus*, and policing is known to occur in other bacterial cooperation such as with quorum-sensing cheats among *Pseudomonas aeruginosa.* Here, bacteria synthesize cyanide and the cheats are unable to produce the enzyme to degrade it (Wang et al. 2015).

Though not in service of cooperation, Cao et al. (2015) also find that motile *M. xanthus* cells are inhibited from social motility when mixed with nonmotile cells. The inhibition was also found to be Tra-dependent. The authors suggest that the nonmotile cells kill the motile ones by a Tra-dependent mechanism. A possible means for this is expression of a toxin and antitoxin pair possessed by the nonmotile cells with the toxin delivered to the motile cells by OME. So policing may offer an explanation for the stability of cooperation in the face of potential developmental cheats. Here, the explanation rides on the ability to eradicate cheats through the aforementioned mechanisms. Table 2 summarizes the proposed explanations for cooperation in *M. xanthus* quorum sensing, motility, and fruiting body formation. In the next section, we will address whether these explanations, among others, can account for *M. xanthus* cooperative predation.

**Table 2:** Summary of proposed explanations for cooperation in *Myxococcus xanthus*.

Kin selection	Signaling	Punishment
Quorum sensing	Motility	(Possibly) Motility
Fruiting body formation	Fruiting body formation	Fruiting body formation

## Explaining Wolf-Pack Predation in *Myxococcus xanthus*

It is widely believed that *M. xanthus* cooperate in predation, yet *M. xanthus* do not display many of the typical predation mechanisms of other bacteria, for example, the use of diffusible antibiotics seen in the *Streptomyces* species and they do not invade the cell membrane of prey, as seen in *Bdellovibrio bacteriovorus* (Berleman and Kirby 2009). Rather, their predation involves the secretion of secondary metabolites and hydrolytic enzymes—such as proteases and lysozymes—into the extracellular environment which kill and lyse prey cells. The killed prey produce a pool of hydrolytic breakdown products in the extracellular environment, the uptake of which promote *M. xanthus* cell growth. Their predation has often been referred to as “wolf-pack” predation since, in being a group effort, it resembles the predation seen in other species such as wolves and lions (Pérez et al. 2016; Furness et al. 2020). Though *M. xanthus* occasionally kill via direct contact with prey, communal predation involves secreted factors which are able to migrate, allowing predation to be contact-independent. As a result, *M. xanthus* require proximity to prey since nutrients will be released in the extracellular environment (Berleman and Kirby 2009). There are two important features of their communal predation which differ from many other bacteria and require cooperation. One feature is the active searching of prey using social motility and the characteristic movement *M. xanthus* exhibit when lysing prey colonies.^6^ Another feature is the supposed density-dependence of the activity, requiring a quorum.

Though there is some evidence of single myxobacterial cells being able to lyse prey (Shilo 1970; McBride and Zusman 1996; Berleman and Kirby 2009), group predation offers advantages. Most obviously, there is a higher level of predatory materials secreted or, where predation is contact-dependent, a greater number of attacking cells. Another advantage may lie in having a larger genetic representation, which makes it more likely that the cells have the ability to encode the necessary lipases, proteases, nucleases and other digestive enzymes required for killing and lysing prey (Pérez et al. 2014). As noted earlier, cell diversity exists in *M. xanthus* populations so, with a larger genetic representation, mutants that do not produce the required predatory materials constitute less of population. Yet Pérez et al. (2016) note that, though this social behavior was described 75 years ago, most of the systems by which cooperative predation operates remain to be elucidated. Muñoz-Dorado et al. (2016) also cite many open questions, such as the ecological consequences of *M. xanthus* predation on natural environments, how the prey induces the predatory enzymes or secondary metabolites in *M. xanthus*, how predators detect prey, and when contact with the prey is necessary for predation.

The open question I will address here is whether we have a good model to explain the persistence of cooperative *M. xanthus* predation in the face of potential developmental cheats. Free riders could benefit from the degradation of prey macromolecules in the extracellular environment without contributing to the development of the secondary metabolites and enzymes needed to kill prey. Empirically, studies have not considered how much this occurs during predation across prey types (especially under natural conditions) relative to contact-mediated killing. However, since free riding is found in other *M. xanthus* activities, it is reasonable to suppose it is a problem that also has to be overcome in predation. If we cannot find an adequate explanation for *M. xanthus* predation, we can draw lessons from the limitations of these kinds of explanations of social phenomena.

### Gaps in our Theoretical Explanations

Can our existing explanations of *M. xanthus* cooperative activity also explain cooperative predation? Not straightforwardly. First, as mentioned earlier, kin selection reliant on limited dispersal will not straightforwardly explain *M. xanthus* cooperative predation. This is because cell diversity exists in *M. xanthus* populations—genetic diversity can be amplified by environmental stresses and mutations. Some spatial assortment will of course occur as a result of fruiting body formation, but even here, we have seen the need for a kin recognition mechanism to overcome the problem of cheating genotypes. Kraemer and Velicer (2011) note that if the mean mutation rate of *M. xanthus* is roughly similar to *Escherichia coli*, any given fruiting body should contain dozens of mutational variants. They also find that some *M. xanthus* genotypes disperse over large distances. Ultimately, a high level of phenotypic and genetic diversity has been documented among cm-scale *M. xanthus* isolates, showing extremely divergent competitive abilities in fruiting body formation (Vos and Velicer 2006, 2009; Kraemer et al. 2010). Thus, diversity within fruiting bodies is high even though it is found to be much lower than diversity among fruiting bodies, mostly deriving from mutation rather than intergroup migration (Kraemer and Velicer 2011).

Pande and Velicer (2018) also note that *M. xanthus* cells retain the ability to reproduce individually, allowing for lower-level selection to undermine cooperative behavior at the multicellular level. In a study on genetic diversity within *M. xanthus* populations, it was found that eight out of ten of the fruiting bodies studies were found to be internally diverse, almost half exhibiting significant variation in social swarming phenotypes and large variation in the number of spores produced (Kraemer and Velicer 2011). It is proposed that even within kin groups, frequency-dependent selection can maintain both cooperators and socially defective cheaters (Velicer et al. 2000). No work has currently been published on the extent to which limited dispersal and spatial assortment can facilitate kin selection in *M. xanthus* predation specifically. However, the lack of such work may indicate that the problem of genetic diversity is too great to allow for a simple limited dispersal explanation.^7^

Perhaps kin recognition can overcome the difficulty of within-group variation? The major mechanism to facilitate kin recognition is OME. Here, goods are exchanged only after the involved parties are verified in a cell contact–dependent manner. This system contrasts with what occurs in predation, where secreted public goods are poorly controlled once they leave the cell boundary and bystanders can take advantage of the products. In other words, we cannot appeal to mechanisms that ensure kin recognition in a contact-dependent manner when the fruits of cooperation are in the extracellular environment during predation. This is especially true when we note that OME-dependent kin recognition occurs in nutrient-starved environments, since this is when *M. xanthus* transitions to a multicellular fruiting body, whilst predation occurs in nutrient-rich environments. Further work would need to be done on the extent to which kin recognition is required for positive assortment among *M. xanthus* and whether there are any other mechanisms of kin recognition among *M. xanthus* which do not require cell–cell contact.

Second, signaling may not be a good explanation of *M. xanthus* cooperative predation as it is possible that *M. xanthus* predators are not signaling to one another during predation but rather individually responding to cues from prey. Some have suggested that *M. xanthus* does not make quorum-sensing chemicals, AHLs (N-acyl homoserine lactones) itself, but that they are produced by potential prey organisms, stimulating predatory activity. This is sometimes referred to as “eavesdropping” on prey conversations (Furness et al. 2020; Kaimer et al. 2023). As such, it is not clear whether quorum sensing is part of cooperative predation; *M. xanthus* cells could be individually reading off the cues of prey cells rather than signaling to one another. Of course, this is not to say conclusively they could not be signaling to each other.

Indeed, one way we might explain the stability of group predation is that positive assortment might be secured by the cell–cell signaling involved in motility. If *M. xanthus* cells signal to one another to ensure social motility, then it is possible this ensures they are already among kin where the free-rider problem no longer arises and kin selection may therefore explain cooperative predation. This is related to the discussion in the next subsection—part of the reason we may not have come to adequate explanation of *M. xanthus* cooperative predation is that we have been considering this behavior in isolation. It is possible that cooperation in one sphere affects downstream cooperation in another. Furthermore, it is worth noting that an upstream explanation in one does not necessarily mean we have an explanation for all resultant cooperative activities. The game being played and the particular free-rider incentives will differ in each of the cooperative activities of *M. xanthus* and thus different explanations might be required to account for the stability of cooperation.

Finally, though some policing mechanisms exist in other areas of *M. xanthus* cooperation, there is little evidence of punishment for cheating in *M. xanthus* predatory activities. It has been suggested that in isogenic strains, prototrophs killed their auxotroph siblings via the T6SS—a multicomponent protein secretion system popular in Gram-negative bacteria which punctures and delivers a range of effectors that damage cells (Troselj et al. 2018). However, this was only observed under limited conditions (Kaimer et al. 2023). Not only this, but this form of punishment is also contact-dependent. As such, it would offer an only partial explanation of stability in cooperative predation where bystander cells can take advantage of prey macronutrients. The same is true of Rhs proteins which signal bind and deliver C-terminal toxins to neighboring cells (Kaimer et al. 2023). In order for punishment or policing to account for *M. xanthus* cooperative predation, further laboratory studies would need to be conducted on cheating genotypes in predatory contexts. We would need to understand to what extent such policing mechanisms are used during group predation and whether contact-dependence is a problem for policing. Indeed, as mentioned in the previous paragraph, if signaling involved in social motility ensures *M. xanthus* are already among kin, there might not arise the need for policing mechanisms, or such policing mechanisms might be able to be delivered via contact in swarms with close proximity.

So, it seems, the theories put forth to explain cooperation in other areas of the *M. xanthus* life cycle—signaling, punishment, and kin selection—do not yet offer satisfactory explanations of *M. xanthus* cooperative predation in the absence of further empirical research. Further, there is the question of how explanations put forth in other areas of *M. xanthus* cooperation interact with potential explanations of *M. xanthus* predatory activity. The current discussion demonstrates the need for these explanations to be supplemented by further biological realism and mechanistic detail.^8^ We might also extend our theories under consideration. It will be obvious that explanations that appeal to cultural forms of selection or reputational forms of selection will not be applicable among bacteria, so I exclude those from consideration. In what follows, I will examine whether reciprocity, commitment, or group selection can properly account for *M. xanthus* cooperative predation.

Trivers (1971) offered an explanation of altruistic behavior toward nonkin in his theory of reciprocal altruism. Broadly, individuals pay a current cost for the benefit of a social partner’s reciprocation. However, this does not appear to be the most apt explanation of *M. xanthus* cooperation as there is no dividing of the good in order for reciprocal exchange in the form of reciprocation at a later date or in the immediate interaction. In other words, reciprocal altruism is a good explanation for cooperation in dyadic interactions over time, but not in simultaneous-move public goods games.

Schelling (1960) introduced commitments in his work on bargaining and conflict in game theory. For Schelling, commitments are strategic moves which induce the other player to choose in one’s favor. They do so by altering the attractiveness of the options for the committed actor, thereby changing the optimal option for both sender and receiver. However, commitment requires a pre-play signal which introduces a cost to reneging in order to incentivize cooperation. As mentioned earlier, there is debate about whether there is truly pre-play signaling in predation in the form of quorum sensing. As such, we may not have a pre-play signal. Nor do we have a cost to reneging. As mentioned earlier, there is some nascent evidence of punishment in fruiting body formation and in social motility, but not in predation. So commitment suffers from the same explanatory gaps in accounting for the stability of *M. xanthus* cooperative predation.

Under Sober and Wilson’s (1998) theory of group selection, altruism can evolve as different groups make different contributions to the same reproductive pool, from which new groups are formed. If a group with a higher proportion of altruistic actors has greater reproductive output, the resulting proportion of altruists is greater than that of both initial groups combined. Group selection remains a live option for explaining for *M. xanthus* cooperative predation. However, the relative importance of group selection has been contested over its history^9^ and its application to *M. xanthus* predation would require empirical evidence which we do not currently available. We would need to show that *M. xanthus* populations were subdivided into competing groups over their evolutionary history, that there are mechanisms that sustain between-group variation, and mechanisms that affect a group’s proliferation. In particular, there does not seem to be a group-level property in predation that is meaningful for fitness. By contrast, the division of labor in fruiting body formation could be seen as a group-level property with fitness consequences—altruistic cells undergo programmed cell death in order to release nutrients that assist in the morphogenesis of rod-shaped cells into spores, increasing sporulation efficiency.^10^ However, there is no such analogy in the case of predation. The question of what secures cooperative predation is thus open. Again, this is not to say our major theories cannot account for it, but rather, in order to do so, much more biological realism and mechanistic detail is required to understand and explain the social phenomena. In the following two subsections, I present some difficulties which I believe shed light on why we have thus far failed to come to an answer on this question.

### Mischaracterizing the Problem

One reason we may not have come to an adequate explanation for *M. xanthus* cooperative predation is that we may have misrepresented the game under consideration. It is frequently assumed that *M. xanthus* cooperative predation is well-characterized by a linear public goods game (Cao et al. 2015). In this game, multiple actors have the option of contributing some of their endowment to a public pool. This sum is then multiplied and split among the actors. The group’s total payoff is highest when every actor contributes all of their endowment. However, the Nash equilibrium in this game is simply zero contributions by all. Each actor would do best by not contributing anything and free riding off the contributions of others. In the myxobacteria case, the endowment is the digestive enzymes and secondary metabolites used to kill and lyse prey. The contribution concerns the amount of each of these produced by an individual cell, which involves a cost to the cell. The return is the prey nutrients. Here, we need an explanation for how agents overcome the incentive to free ride and contribute nothing. However, it is possible that this is not what is going on in *M. xanthus* cooperative predation. Perhaps this phenomenon is better represented by a nonlinear public goods game or threshold public goods game. In a linear public goods game, some form of positive assortment or enforcement is needed to ensure that cooperation is maintained. However, Archetti (2018) argues that biological public goods should be modelled using a sigmoid or step function rather than a linear or concave function. Archetti and Scheuring (2016) argue that, in the case of diffusible molecules, nonlinearities arise because the effect of biological molecules on cell fitness is typically a sigmoid function of their concentration due to threshold dosage effects or due to the cooperative binding of ligands (Cornish-Bowden 2012; Frank 2013; Zhang et al. 2013). In social interactions, nonlinearities arise when just a portion of the group is large enough to detect predators or take down difficult prey (Pulliam et al. 1982; MacNulty et al. 2011). Further empirical evidence for nonlinear public goods games can be found in *Escherichia coli* production of antibiotic resistance molecules and in the production of pyoverdine in *Pseudomonas putida* (Chuang et al. 2010; Becker et al. 2018). Smith et al. (2010) specifically showed the positive effects of non-additivity in sporulation on *M. xanthus* cheater resistance.

In a nonlinear public goods game, cooperation can often be maintained endogenously. Here, cooperators and defectors stably coexist in equilibrium when the cost/benefit ratio of cooperation is below some critical value (Archetti and Scheuring 2011, 2012; Wang and Chen 2019).^11^ Increasing the inflection point of the sigmoid function increases the stable proportion of cooperators (Archetti and Scheuring 2011, 2012).^12^ The steepness of the curve affects the ability to achieve polymorphic equilibrium at higher costs of cooperation, yet even for an extremely shallow sigmoid function, a mixed equilibrium can be maintained unless the cost of cooperating is relatively high (Archetti and Scheuring 2011, 2012). If nonlinearity is a true characterization of *M. xanthus* cooperative predation then direct benefit to the cooperator would be the explanation for the persistence of cooperative predation and, unlike in a linear public goods game, mechanisms for relatedness, signaling or punishment are not required to sustain cooperation.

A threshold public goods game is one version of a nonlinear public goods game. In a threshold public goods game, the public good is not produced at all unless some minimum level of contributions is met. If this is so, the opportunity for free riding only arises if there is redundancy. If it happens to be the case that there is just as much production of predatory materials as is needed to kill and lyse prey cells, each individual cell is contributing for their direct benefit. In other words, if there are exactly *k-1* cooperators in the rest of the group, where *k* is the minimum threshold level for successful production of the public good, being the *k*th cooperator is personally beneficial. If the actor did not contribute, the public good would not be produced and so the individual actor would suffer, unlike in the traditional public goods game. Here, cooperation is sustained at the group level simply because it is required for any benefit to be received by the individual actor. As such, in any nonlinear public goods game, cooperation can be maintained without need for positive assortment or enforcement mechanisms.^13^

This is not to say that cooperation could not be maintained via, for example, kin selection. Of course, there is nothing in the theory of kin selection that requires its benefits be linear.^14^ Rather, I mean to say kin selection—or another means of positive assortment—is not *required* to sustain cooperation in a nonlinear public goods game. In these games, cooperation can be maintained endogenously without any need for positive assortment or punishment. In other words, when we move from a linear to a nonlinear public goods game, we move from the problem of altruism in an *n-*player Prisoner’s Dilemma to the problem of mutual benefit. The explanation for the stability of mutually beneficial cooperation is direct benefit to the cooperator.

Alternatively, the problem may lie in the fact that simple game-theoretic characterizations of cooperative problems—in public goods games, Prisoner’s Dilemmas, or Stag Hunts—model selection on individual strategies rather than on genes that influence multiple strategies. Indeed, we know that the predatory ability of *M. xanthus* is negatively affected by mutations that are implicated in the early stages of development (mutations in *asgA*, *asgC*, *asgE*, *sdeK*, and *csgA*), as well as in genes of the chemotactic *frz* system, which modulates motility and development (Pham et al. 2005; Berleman et al. 2006, 2008). Previously, we have considered these interactions separately and offered independent explanation for cooperation in these interactions. However, it is possible that *M. xanthus* cooperation is better modelled using strategies which crosscut games. This would change our analysis of the explanation. Cooperation in other areas of the developmental cycle will have implications for downstream cooperation in other areas. The free-rider problem may not arise in predation if, for example, gene expression for the production of predatory materials was systematically linked to that of development in fruiting body formation, which is purportedly explained by a mixture of kin selection, signaling, and punishment.

So, one way in which we might better understand the stability of *M. xanthus* cooperative predation is by focusing our empirical and theoretical research on the mechanistic detail required to inform precise formal modelling. By better understanding the conditions under which cooperative predation is maintained and the mechanisms which ensure this, we will be able to understand if it is best represented by a public goods game, and if so, what type. This will in turn affect whether we must appeal to theories of positive assortment or enforcement in order to explain the stability of cooperation. In other words, by setting up the problem precisely, we would narrow appropriate avenues for explanation.

### The Importance of Ecological Context

Another potential reason why we have not come to an adequate explanation for *M. xanthus* cooperative predation is that our focus on cooperative models might be inhibiting us from considering the ecological context in which cooperation happens. Tarnita (2017) argues that cooperative models can come at the expense of (1) consideration of ecological context, (2) alternative hypotheses that might reveal different roles and different types of interactions between the purported cooperators and cheaters, or (3) it can obscure the possibility that cooperation or cheating are not ends in themselves but are transient phases of behavior. Here, I elucidate her ideas and support these with further research.

In regard to (1), ecological context has often been missing in studies of myxobacteria due to the laboratory set up. Tarnita (2017) notes that ecology is the main determinant of the type of emerging group formation and social organization among many organisms. These ecological considerations could be availability of breeding habitats, intraspecific competition, spatiotemporal environmental variation, altitude, and other factors. Yet cooperative models which seek to explicate in vitro studies of *M. xanthus* predation would not capture this. Indeed, it has been found that other bacteria behave differently in competitive contexts in structured versus unstructured environments. Alellopathy refers to the production of toxic compounds by bacteria that kill competitors and the success or failure of this mechanism depends on the amount of toxin produced as well as the structural environment. In an unstructured environment such as a well-mixed liquid culture, toxin producers are eliminated at low frequency (Chao and Levin 1981). However, in a structured environment such as an agar plate, cells that produced the toxin had an advantage even at low frequencies since the structure allows the local frequency of toxin producers to be higher than their global frequency, thereby benefiting from local competitor death (Grieg and Travisano 2008; Chao and Levin 1981; Weiner 2000).

On (2), that alternative hypotheses might reveal different roles between cooperators and defectors depending on genotype and environment, Tarnita (2017) cites a study about pyoverdin produced by bacteria from the genus *Pseudomonas.* Pyoverdin is an iron-chelating molecule; however, predications about whether pyoverdin can be exploited by cheaters depend strongly on both genotype and environment. What she intends to show is that bacteria are not simply cooperators and defectors—there may be sensitivities to the environment and genotype that changes their expression of cooperation or defection. Rainey et al. (2014) write that pyoverdin production might conform to a social dilemma under one set of conditions, but can change entirely as a result of small changes in the nutritional status of the environment. Not only this, but among *M. xanthus*, Schaal et al. (2022) produced disruptions of the gene *csgA* in diverged genomic backgrounds. The genomic background refers to the genotype of all genes that may interact with the gene of interest, and can therefore affect the specific phenotype. They demonstrated that whether a mutation generates a cheating genotype will depend on its genomic context. Note that this would also limit the ability of any given cheating mutation to spread by horizontal gene transfer across different genomic backgrounds. So our cooperative models are not fine-grained enough to consider genotype–environment interactions and genomic context. As a result, it would be mistaken to label *M. xanthus* cells as cooperators or defectors without consideration of when this expression is realized and the underlying ecological context.

Finally, in (3), Tarnita (2017) suggests cooperation and cheating are not ends in themselves but could represent a transient phase in myxobacteria evolution. In *M. xanthus* sporulation, only a minority of cells survive, therefore limited sporulation provides a fitness advantage. A study by Fiegna et al. (2006) looked at one cheater that fails to produce viable spores in monoculture but, in mixtures with a proficient strain, sporulates even more effectively than the proficient genotype. It therefore relies on the presence of a proficient host to avoid extinction during starvation. In laboratory experiments, this cheater was allowed to compete against the socially proficient strain in development on starvation agar and then growth in a liquid medium. It eventually mutated into a novel social type. Descendants of the cheater strain re-evolved the ability to sporulate independently and overtook the previous cooperative population in the mixed culture, also demonstrating an additional advantage of growth in a liquid medium. Dominance of the new strain is demonstrated by its ability to sporulate independently and its ability, at high frequencies, to hinder the efficiency of the sporulation of the previous socially proficient strain. This again suggests that our cooperative models are not fine-grained enough. The evolution of new strategies can be rapid enough that this should not be excluded from our models.^15^

It is important to note that ecological context may also play a role in a mischaracterization of the game at hand. Not only may environment or gene–environment interactions change an organism’s expression of cooperation or defection but they can change the game being played. As an illustrative example, consider the hunting strategy of a pack of animals in an environment which is heavily wooded versus the hunting strategy of a pack of animals where the environment is barren. In the former, ambush hunting may be more likely and, in the latter, persistence hunting. The number of actors, the strategies and free-rider incentives will all vary with features such as these.

To return to the myxobacterial case, it is first worth noting that positive density dependence is one major component supporting the view that microbial behaviors are cooperative. For example, this correlation is shown in the strong positive density dependence S-motility and of spore production. However, the situation is more complicated in the case of predation. The effects of density on predation can be highly contingent on the prey environment, i.e., what species of prey is being consumed. In some prey environments, density seems to have very little or no effect on predatory growth whereas in others it seems to have a clear effect.^16^ This supports the view that the degree to which and manner in which myxobacterial predation is a cooperative enterprise will depend on abiotic and biotic ecological factors. Again, the importance of ecological context is not limited to discussion of *M. xanthus* predation rather than their other cooperative activities, but focusing on this one difficult-to-explain case is instructive for how we ought to examine other social phenomena.

## Conclusion

*Myxococcus xanthus* cooperate in a number of different ways. Some explanations have been proposed for how cooperative stability is maintained in the face of potential developmental cheats who do not contribute. However, I argue that none of our major existing explanations seem to neatly apply to *M. xanthus* cooperative predation, and there is good reason for further exploration. I then suggested why we might have failed to thus far account for the cooperative predation of *M. xanthus.* One issue might be a mischaracterization of the cooperative phenomenon as a traditional public goods game rather than a nonlinear public goods game, or a mischaracterization of selection as operating on strategies rather than genes which influence multiple strategies. Another reason we might have failed to account for *M. xanthus* cooperative predation is in not considering ecological and genomic context: either the natural environment, the interaction of genotype and environment, or the potential for transient phases of cooperation or defection. Many of our major models for the evolution of cooperation are too coarse-grained to capture this nuance.

Three upshots follow from this work. First, *M. xanthus* predation requires greater empirical attention. In particular, in order to better address how this practice is stable, studies will need to look at under what circumstances *M. xanthus* engage in individual versus collective predation, whether signaling precedes cooperation, whether there is punishment for defection from predatory activity, to what extent disruption of genes involved in other areas of cooperation such as fruiting body formation causes disruption of predatory capacities, whether there is a threshold level of contribution of effective killing and lysing of prey cells, how *M. xanthus* behave in their natural environment, and whether the conditions for group selection are met over their evolutionary history. The answers to these questions will help our understanding of how to model this cooperative phenomenon precisely as well as how to answer the question of how cooperation is sustained. Second, and relatedly, a new model to represent predation could lead to new avenues for explaining how *M. xanthus* cooperation is stable. *M. xanthus* may be better represented by a nonlinear public goods game, threshold public goods game, or a composite game. If represented by a nonlinear or threshold public goods game, individual benefit would offer an explanation for the stability of cooperation. In a composite game, cooperation in predation would depend on cooperation in other areas of the *M. xanthus* life cycle.

Third, and most importantly, this analysis provides insight into when and where our simplified cooperative models can provide good explanations of real-world behaviors. I have used *M. xanthus* as a case study as it is useful for understanding the evolution of cooperation generally. *M. xanthus* exemplifies the transition to multicellularity and exhibit multifaceted and flexible cooperative practices. However, this is not to say the analysis here only applies to *M. xanthus* cooperation or only to cooperation among bacteria. Indeed, in discussing the importance of ecological context in myxobacteria studies, Tarnita (2017) appeals to a number of examples from the animal kingdom, showing the pervasive importance of ecology in understanding cooperation. In particular, she cites the fact that spatiotemporal environmental variation and lack of available breeding habitats are important drivers of cooperative breeding in birds (Emlen and Wrege 1989; Rubenstein 2011). Dispersion and availability of food as well as patterns of predator-avoidance behavior are important factors determining the group size and social organization of African antelope species (Jarman 1974). Intraspecific competition can lead to associations between unrelated ant queens (Ross and Keller 1995; Bernasconi and Strassmann 1999). Altitudinal gradients have effects on length of the breeding season which can determine whether different species of hymenoptera develop a social or solitary lifestyles (Kocher et al. 2014). Our theories on the evolution of cooperation, in abstracting away from these details, are only approximations of the mechanisms that sustain cooperation and are not generalizable across different contexts. The same is true of a possible mischaracterization of the cooperative phenomenon at hand—this applies equally well to cooperation across the animal kingdom.

Again, the case study on *M. xanthus* predation is simply illustrative of a broader point—that there are difficulties in applying coarse-grained, abstract, how-possibly theories of the evolution of cooperation to real-world social phenomena. I have sought to draw out two of these difficulties—the potential for mischaracterization of the problem and the importance of ecological and genomic context—using *M. xanthus* cooperative predation as an illustrative case. *M. xanthus* represents a model system in studying the evolution of cooperation since it exhibits transitory multicellularity in response to environmental conditions and cooperates flexibly in multiple spheres of its developmental life cycle. Yet, what explains the stability of its cooperative predation is not yet well-understood. I have shown why our major theories of the evolution of cooperation may not apply neatly to this social phenomenon. This is not to say one of these theories cannot be the explanation of *M. xanthus* cooperation in predation, but rather, that there are difficulties in applying the theories that are salient in this case. This should prompt us to consider such difficulties in the application of these theories to other social phenomena as well.

This is far from saying these major theories of the evolution of cooperation do not have uses. They lay the theoretical groundwork for empirical study. It is only after we have come to theoretical answers such as kin selection that we may examine to what extent this is a driver of cooperation in the real world. However, caution is needed not to be tempted by the cleanness of the theory and replace empirical precision in our studies of real-world behavior. Empirical studies that seek to validate these theories should pay close attention to the characterization of the game at hand, to the natural environment of organisms, the interaction of genotype and environment, the genomic context, and the potential for transient phases of cooperation or defection. They should be supplemented with biological realism and mechanistic detail. A more integrated approach to explain cooperative behavior would look to ecological evolutionary developmental (eco-evo-devo) biology. The field integrates concepts such as developmental symbiosis, plasticity, genetic accommodation, extragenic inheritance, and niche construction (Gilbert et al. 2015). An approach that integrates function and mechanism might guide our explanatory search in a more robust way than the current, idealized focus on function seen in our major theories. Lastly, it is important that our search for greater mechanistic detail should not be informed by our need to fit it into the framework of one of our major theories of the evolution of cooperation. Rather, with greater detail, we may narrow down our avenues of explanation.

## Footnotes

1 See Forber and Smead (2015) for a criticism of this classification.

2 The extent to which these are differing theories has been debated. See Lehman and Keller (2006) on integrating direct benefits, information sharing, kin selection, and the greenbeard effect into one conceptual framework. See also work showing that group selection is mathematically alike to the theory of kin selection (Frank 1995; Henrich 2004; Okasha 2005; West et al. 2007; Marshall 2011).

3 West et al. (2007) for discussion.

4 Some have contested the importance of rippling for predation or even coordination in rippling behavior (Berleman et al. 2008; Berleman and Kirby 2009).

5 Note that to punish imposes a cost on an agent but benefits the group, so punishment is itself an altruistic behavior in need of explanation. This is known as the second-order problem of altruism (Boyd et al. 2005). For the purpose of brevity, I will not discuss this here, though several solutions have been proposed (Henrich and Boyd 2001; Sasaki and Uchida 2013; Ozono et al. 2016).

6 Berleman and Kirby (2009) found that though direct movement toward casitone-yeast extract mixtures was observed in *M. xanthus* colonies, individual cells were not found to exhibit directed movement (Berleman and Kirby 2009). See also Taylor and Welch (2008).

7 Although this may provide an explanation for cooperation in other bacteria in their natural environments, for example, *Bacillus subtilis* (Belcher et al. 2023).

8 I thank an anonymous reviewer for this suggestion.

9 For an overview see Okasha (2001).

10 See also Hudson et al. (2002) on fecundity and dispersibility increasing as a fraction of cells which perform an altruistic function in cellular slime molds.

11 For an analytic proof of the existence of the mixed equilibrium, see Archetti (2018).

12 Changing the position of the inflection point and the steepness at the inflection point (reflecting the synergistic effects of volunteering), allows us to recover the linear public goods game (or *n*-person Prisoner’s Dilemma) and the Volunteer’s Dilemma (or *n-*person Chicken or Hawk-Dove) as special cases. In particular, when the steepness at the inflection point tends to 0, the public good is a linear function of individual contributions and, when it tends to infinity, the public good is a step function of individual contributions.

13 The issue of mischaracterization is further complicated by the fact that we may believe there are public goods games where they do not exist. It is possible that the phenomenon is not social at all. Indeed, Rainey et al. (2014) note that there are a number of testable predictions of a public good hypothesis that will help us to establish its existence: nonproducers gain advantage in the presence of the secreted product, nonproducers will do poorly in the absence of the producers, and producers would do poorly in the absence of nonproducers. Nonetheless, that evidence is consistent with public goods production does not necessarily mean the underlying trait is cooperative (Driscoll et al. 2013). Public goods are not simply “extracellular metabolites” (Rainey et al. 2014). Instead, one might argue it further needs to be shown that public good production is selected or maintained because of a benefit conferred to recipient (West et al. 2007). With myxobacteria, this task becomes much harder as it requires us to look at the ecological circumstances under which a given trait evolved or is maintained (see also discussion in the following subsection). Experiments concerning public goods production and cooperation might be of limited use because of the scientists’ choice of medium, density of bacteria, ratio of competitors, and the specific details of competing genotypes, which will often fall short of the correct ecological conditions given our limited information (Rainey et al. 2014).

14 See Queller (1992) for a generalization of kin selection theory.

15 One way of overcoming this is to interpret actors within the model as not one of either cooperating or defecting *types* but of each having either a probability of cooperating or defecting. Indeed, this is a common interpretation employed in evolutionary game theory rather than classical game theory. However, the issue still remains that our models of the evolution of cooperation may be too coarse-grained to capture the nuance of ecological and genomic context as well as the evolution of new strategies.

16 In conversation with Greg Velicer, investigations ongoing (personal communication, 21 May 2024).
